# Screening strategies and laboratory assays to support *Plasmodium falciparum* histidine-rich protein deletion surveillance: where we are and what is needed

**DOI:** 10.1186/s12936-022-04226-2

**Published:** 2022-06-24

**Authors:** Khalid B. Beshir, Jonathan B. Parr, Jane Cunningham, Qin Cheng, Eric Rogier

**Affiliations:** 1 Faculty of Infectious Diseases, London School of Hygiene and Tropical Diseases, Keppel Street, London, WC1E 7HT UK; 2 Division of Infectious Diseases and Institute for Global Health and Infectious Diseases, University of North Carolina at Chapel Hill, Chapel Hill, NC 27599 USA; 3 Global Malaria Programme, World Health Organization, Geneva, Switzerland; 4 Drug Resistance and Diagnostics, Australian Defence Force Malaria and Infectious Disease Institute, Brisbane, Australia; 5 QIMR Berghofer Medical Research Institute, Brisbane, Australia; 6 Division of Parasitic Diseases and Malaria, Center for Global Health, Centers for Disease Control and Prevention, Atlanta, GA 30029 USA

**Keywords:** Malaria, Rapid diagnostic test, *pfhrp2*, *pfhrp3*, Gene deletions, Laboratory assay, Surveillance, Histidine-rich protein

## Abstract

Rapid diagnostic tests (RDTs) detecting *Plasmodium falciparum* histidine-rich protein 2 (HRP2) have been an important tool for malaria diagnosis, especially in resource-limited settings lacking quality microscopy. *Plasmodium falciparum* parasites with deletion of the *pfhrp2* gene encoding this antigen have now been identified in dozens of countries across Asia, Africa, and South America, with new reports revealing a high prevalence of deletions in some selected regions. To determine whether HRP2-based RDTs are appropriate for continued use in a locality, focused surveys and/or surveillance activities of the endemic *P. falciparum* population are needed. Various survey and laboratory methods have been used to determine parasite HRP2 phenotype and *pfhrp2* genotype, and the data collected by these different methods need to be interpreted in the appropriate context of survey and assay utilized. Expression of the HRP2 antigen can be evaluated using point-of-care RDTs or laboratory-based immunoassays, but confirmation of a deletion (or mutation) of *pfhrp2* requires more intensive laboratory molecular assays, and new tools and strategies for rigorous but practical data collection are particularly needed for large surveys. Because malaria diagnostic strategies are typically developed at the national level, nationally representative surveys and/or surveillance that encompass broad geographical areas and large populations may be required. Here is discussed contemporary assays for the phenotypic and genotypic evaluation of *P. falciparum* HRP2 status, consider their strengths and weaknesses, and highlight key concepts relevant to timely and resource-conscious workflows required for efficient diagnostic policy decision making.

**Supplementary Information:**

The online version contains supplementary material available at 10.1186/s12936-022-04226-2.

## Background

Malaria caused by *Plasmodium* species has plagued humanity and shaped the human genome for millennia. However, identification and visualization of this parasite to confirm infection did not occur until the late nineteenth century with advances in microscopy and staining techniques. Widespread deployment of antigen-detecting malaria rapid diagnostic tests (RDT) commenced in the first decade of the twenty-first century as a result of the World Health Organization (WHO) policy change requiring parasitological confirmation of malaria infection in all age groups prior to administration of anti-malarials [1–4]. RDTs have proved to be a great asset for malaria diagnosis, case management, and epidemiological surveillance, and multiple *Plasmodium* antigen targets exist for parasitological diagnosis of blood-stage infection [5]. At present, the most sensitive and specific RDT target is *Plasmodium falciparum* histidine-rich protein 2 (HRP2), which is abundantly expressed and released by merozoites during blood-stage infection [6, 7]. Due to the predominance and clinical importance of *P. falciparum* in sub-Saharan Africa and limited access to quality microscopy, many countries currently utilize RDTs detecting only HRP2 as their primary malaria diagnostic test [8]. The histidine-rich protein 3 (HRP3) antigen is paralogous to HRP2 and encoded by the *pfhrp3* gene on chromosome 13, whereas *pfhrp2* is found on chromosome 8 [9, 10]. Both genes are found near the subtelomeric regions [11], where recombination occurs commonly. Though many of the same epitopes for monoclonal antibodies are also found within HRP3 [12], this protein is considerably shorter in length and by itself contributes to a positive RDT result only at higher parasite densities [13].

Deletion of the *pfhrp2* gene was first identified in culture adapted parasites, but it was thought those mutants would be unable to establish human infection viably in natural settings. It was of great surprise when a large percentage of *P. falciparum* isolates collected in Peru from 2003 to 2008 lacked *pfhrp2* and/or *pfhrp3* (*pfhrp2/3*), where HRP2-RDT use was basically non-existent [14].This high prevalence of gene deletions led the WHO to recommend using tests targeting alternative antigens or quality microscopy for malaria diagnosis in Peru, and to urgently map gene deleted parasites in neighboring countries and others outside South America [15]. Since this Peru report was published in 2010, *P. falciparum* populations with deletions of *pfhrp2/3* have been identified in different areas throughout the malaria endemic world, and concerningly, in many countries relying heavily on HRP2-based RDTs [16](apps.who.int/malaria/maps/threats). However, the prevalence of these deletions have only been severe enough to recently change the malaria diagnostic strategy in Eritrea, Djibouti, and Ethiopia in accordance with the WHO recommendations listed below. Previous findings of *pfhrp2* deletions in Peru, Brazil, Colombia, Suriname had also caused these countries to preemptively consider RDT targets other than HRP2 for *P. falciparum* diagnosis [16]. With > 99% of clinical malaria occurring in sub-Saharan Africa due to *P. falciparum,* and the very limited number of alternative diagnostics, compelling evidence is required to justify a change in current HRP2-based RDT diagnostic policies.

The WHO currently recommends that countries alter their malaria diagnostic policies if the local prevalence of *pfhrp2* deletion causing false-negative RDT results in symptomatic *P. falciparum* infection exceeds 5% [17]. For most *P. falciparum* only settings, this change would mean moving from an HRP2-based single-antigen RDT to a single parasite lactate dehydrogenase [Pan-pLDH] RDT, *P. falciparum*-specific pLDH (Pf-pLDH) RDT, or a multi-antigen test (HRP2 plus Pan-pLDH and/or Pf-pLDH). Though a finding of high deletion prevalence would make the decision to move away from HRP2-only RDTs more straightforward, the more common scenario that has been observed is low and mostly geographically heterogeneous prevalence of *pfhrp2* deletion in symptomatic infections [18–24], with seasonal fluctuations [25]. For these increasingly common scenarios, more intense sampling is required to obtain accurate and precise prevalence estimates at the country-level for decision making purposes. Furthermore, nationwide RDT change is a highly challenging process involving recall and/or destruction of RDTs, selection and distribution of alternative quality RDTs may be more difficult to acquire due to limited number of suppliers and increased costs, re-training health care workers, continuous performance surveillance, and other logistical challenges [26]. Thus, the decision to change RDTs should not be made lightly and must be informed by accurate, timely surveillance data and precise prevalence estimates of *pfhrp2*/3 deletions causing negative HRP2-RDTs.

**Fig. 1:**
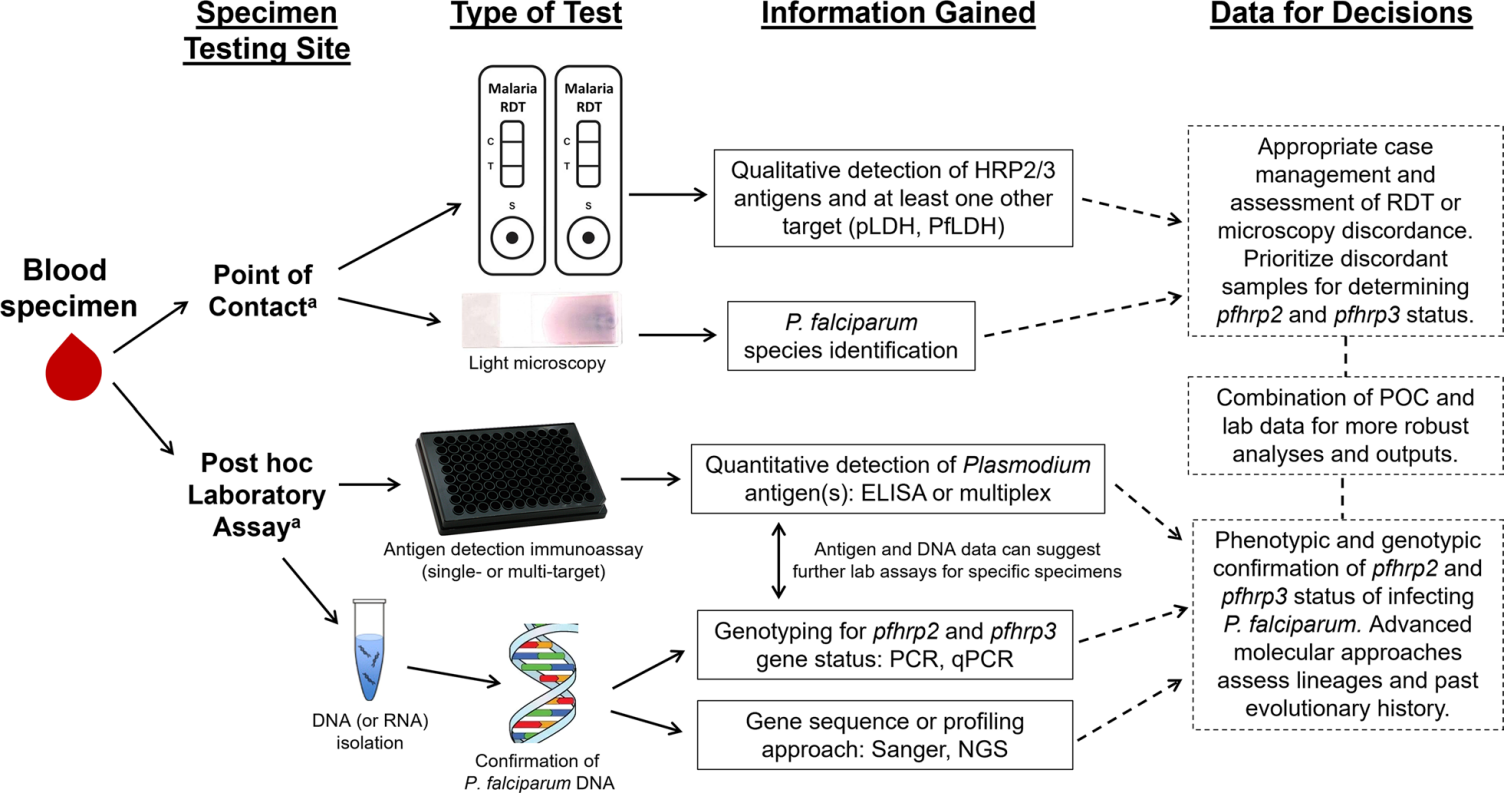
Flow diagram for phenotypic and genotypic testing for confirming *pfhrp2* and *pfhrp3* gene status. Following collection of blood sample, tests can be performed immediately or later by laboratory assays. Antigen and gene data can be gathered for appropriate interpretation decision making. ^a^Point-of-contact (POC) assays will require whole blood, whereas laboratory assays can accommodate whole blood, fractionated blood, or dried blood on filter paper as appropriate starting sample types

In an attempt to improve the quality of *pfhrp2/3* deletion surveillance and establish a degree of uniformity in reporting, the WHO published standard survey protocols to guide country malaria control programs in 2019 [17]. The protocol includes recommendations for well-defined survey populations, standardized sampling methods, ethical considerations, and technical procedures to confirm deletions and estimate prevalence. These recommendations and the growing consensus in the field confirms the importance of rigorous laboratory methods to confirm deletions. A set of recommendations for accurately reporting gene deletions was published in 2014 [27] and provides technical guidance for laboratory analysis of gene deletions. While these recommendations improved the quality of laboratory results [16], a revision and expansion of these recommendations is planned in response to new technologies and methods established and increasingly used in recent years. Outlined here currently are available lab assays utilized for confirmation of *pfhrp2/3* deletions and consider how they may be best utilized in efforts for data-based decision making regarding HRP2 diagnostics. A summary of types of data collection and testing workflows for assays listed below is presented in Fig. 1.

## Phenotypic screening for the HRP2 antigen

Testing for the HRP2 (and HRP3) antigens in the blood of *P. falciparum* infected individuals can be performed by hundreds of commercially available RDTs using capillary blood from fingerprick at the point-of-care (with results in 15–30 min) or later in a laboratory setting. Current commercial and in-house *Plasmodium* spp. antigen detection laboratory immunoassays include ELISA, chemiluminescent assay, and bead-based assay [7]. All three assays can be applied to multiple biospecimen types in a high-throughput format to allow practical data collection for hundreds of blood samples in a standard workday. The analytical sensitivity of the best-in-class conventional HRP2-RDTs is estimated around 1.0 ng/mL blood [28], but laboratory immunoassays have published limits of quantification below 0.01 ng/mL [7]. While RDTs used in predominantly *P. falciparum* endemic regions may target only HRP2 for detection of this parasite, a limited number also target an additional *P. falciparum* specific antigen, Pf-pLDH, or may include only a single non-specific Pan-pLDH target. Current HRP2 ELISAs remain in single-target format, available chemiluminescent and bead-based assays have the advantage of multiplex formats, allowing for simultaneous detection of HRP2 along with other *Plasmodium* antigens. Mass phenotypic screening of individual biospecimens for the presence/absence of HRP2 can be used as a high-throughput economical tool to provide initial evidence for *pfhrp2* deletions in a *P. falciparum* population by ‘discordance’ with a non-HRP2 target—whether this is an antigen target or some other indicator of *P. falciparum* infection. This approach is particularly useful for very large surveys and in areas where training and reliance on health workers to screen for *pfhrp2/3* deletions using two separate RDTs (HRP2-targeting and Pf-pLDH or Pan-pLDH targeting) or an HRP2 RDT and microscopy is not considered feasible.

Discordant diagnostic profiles in which further characterization is useful are included below:

1. *P. falciparum* blood-stage parasites confirmed by microscopy are expected to have sufficiently high parasite density for detection by quality HRP2-based RDTs [5]. Thus, if *P. falciparum* parasitaemia is visualized on a blood smear but negative by HRP2-RDT, a sample would be labelled as discordant.
2. An RDT detecting HRP2 along with Pf-pLDH (or Pan-pLDH) could provide a discordant result if positive for the pLDH target but negative for HRP2. Depending on the endemic setting, a Pan-pLDH +/HRP2—result could also be interpreted as infection with other *Plasmodium* species.
3. By laboratory assays outside of the point-of-care environment, discordance could be identified by positivity to *P. falciparum* (by previous microscopy, non-HRP2-RDT result, or by PCR positivity) but negativity to HRP2 by laboratory assays. Additionally, if employing a multiplex antigen detection assay, a negative result to HRP2 but positive to other *Plasmodium* target antigens could be labelled as discordant.

Though strong initial evidence for *pfhrp2/3* deletion could be provided by any of the three discordance profiles above, confirmation of gene deletion or loss-of-function mutations requires the use of molecular assays. However, phenotypic characterization using laboratory immunoassays offers the opportunity to evaluate other common causes of RDT discordance, including poor quality RDT performance (poor product quality and/or poor storage conditions), operator error, low parasite densities, or infection with non-falciparum *Plasmodium* spp., among other factors [21]. It should be noted that discordance in test results due to gene deletions is most often caused by parasites with deletion of both the *pfhrp2* and *pfhrp*3 genes. Depending on the antibody used in the antigen screening immunoassay, parasites with deletion of only *pfhrp2* gene (or *pfhrp3* gene) may have sufficient HRP2 or HRP3 antigen to trigger a positive result during phenotypic screening and would not be identified as ‘discordant’. As the main purpose of the surveillance is to determine the rate of false-negative RDT results caused by parasites lacking *pfhrp2/3* genes, antigen screening (especially using HRP2-based RDTs) not only helps to focus on the most relevant samples, but also provides a direct line of evidence on the rate of false-negative HRP2-RDT results in symptomatic patient population.

## Molecular confirmation of *pfhrp2* and* pfhrp3* genes

### Conventional PCR

Conventional PCR is the most commonly used method to confirm *pfhrp2/3* deletions. The wide availability of basic thermocyclers and gel electrophoresis equipment enable its use in diverse laboratory settings. The approach used in the first demonstration of deletions in field isolates from Peru involved amplification of two exons each of *pfhrp2/3* genes, each in a separate reaction, followed by visualization of PCR products on agarose gels [14]. PCR primers and conditions originated from an earlier publication reporting genetic diversity of *pfhrp2/3* genes [29]. Samples that failed to amplify either exon of the *pfhrp2/3* genes were considered a possible *pfhrp2/3* deletion. Because the readout is absence of PCR product, it is critical to demonstrate that there is sufficient and quality parasite DNA in the PCR reaction to produce a product if these genes were present. Quality control for genomic DNA was achieved by amplifying at least two other single-copy genes with similar amplicon sizes to exon 2 of *pfhrp2/3 (*e.g., *msp1* and *msp2* or *glurp*). To complement this approach, others have also used a parasite density cutoff (e.g.,  ≥ 100 parasites/µL) to guard against misclassification of deletions in the setting of low DNA template [30]. Amplification of the genetic sequences flanking *pfhrp2/3* genes has also been used to provide supportive evidence for gene deletions [14, 27]. To date, the conventional PCR method, using slightly varying primer sequences and PCR conditions, has been commonly used to provide laboratory confirmation of *pfhrp2/3* deletions in many studies involving parasite genotypes from around the globe. Pros and cons of this and other PCR methods to detect and classify *pfhrp2/3* genes are summarized in Table 1.

**Table 1:** PCR assays for detecting *pfhrp2* and *pfhrp3* genes with advantages and disadvantages

Advantages	Disadvantages	References
Conventional PCR		Numerous, summarized in: [27, 30]
Can be performed in most labs that can perform molecular diagnosis of malaria	Time-intensive— > 2 h per reaction	
Can readily check presence of flanking genes	Requires multiple distinct PCR reactions; ≥ 6 reactions per sample for control and *pfhrp2/3* genes	
Can detect single and dual exon deletions	Need to visualize PCR products on agarose gels	
	Do not detect gene deletions when gene deleted parasites are mixed with wild-type parasites in the same sample Nested assays are prone to contamination	
	Requires high volume of DNA for > 6 PCR reactions	
Multiplex real-time PCR		[37, 38]
Streamlined workflow	Requires multichannel real-time PCR machine	
Quantitative read-out	Proper interpretation of results requires training	
Can detect mixed infections involving *pfhrp2/3-*deleted and intact strains	Careful optimization required for individual laboratories	
Only one PCR reaction and requires less volume of DNA	May not detect some partial gene deletions involving one exon as most assays targeting one exon only	
Includes internal control		
Digital droplet PCR		[42]
Higher confidence deletion calls than other PCR methods Can detect mixed infections involving *pfhrp2/3-*deleted and intact strains	Specialized equipment that is not widely available Requires advanced laboratory and analysis expertise More expensive than conventional approaches	
Sequencing approaches		[20, 36, 49, 50]
Enable identification of mutations that affect HRP2 expression (e.g., coding changes) Mapping and comparison of deletion breakpoints is possible using next-generation sequencing approaches (amplicon-based and whole-genome) Enables analysis of parasite relatedness, transmission, and evolution	Current approaches are not well-suited for initial deletion identification, especially in lower parasite density samples Specialized equipment that is not widely available Requires advanced laboratory and analysis expertise More expensive than conventional approaches	

Exon 1 of *pfhrp2* and *pfhrp3* genes are prone to spurious amplification, sometimes amplifying exon 1 of the other gene due to a high level of sequence homology in exon 1 between the two genes [30, 31]. It is also challenging to amplify exon 1 efficiently due to AT-rich repeats in the intron; lowering the extension temperature from 72 to 60 °C has been shown to increase PCR efficiency of exon 1 amplification [30]. More recently, a conventional one-step PCR method was established to amplify the full length *pfhrp2* gene in a single PCR reaction [31]. The assay detects deletions of *pfhrp2* that involve either or both exons, overcomes spurious amplification of exon 1 and reduces the number of PCR reactions required. However, the 95% confidence lower limit of detection of this one-step PCR assay is estimated at 133 and 385 parasites/µL from whole blood and dried blood spot field samples, respectively. The detection sensitivity is inferior to conventional PCR that amplifies a single exon [30], likely due to reduced efficiency in amplifying across the highly AT rich intron and producing a longer amplicon.

These experiences confirm the importance of careful assay optimization and quality control within individual laboratories to guard against unintentional misclassification of deletions. Several conditions consistently improve the performance of conventional PCR for *pfhrp2/3* deletion characterization are outlined below:

1. Maximize DNA template in the reaction. Preferably, 5–10 µL of genomic DNA (if using a DBS, extracted from three 6 mm punches) per 25 µL reaction.
2. Utilize a quality hot-start DNA polymerase to increase detection sensitivity and accuracy of gene deletions. As commercial availability of reagents may differ regionally, and each laboratory has inherent subtle differences, the choice of the optimum polymerase should be determined based on internal validation by a lab group.
3. Always include appropriate controls. In addition to the usual no-template control (NTC) and a *P. falciparum* positive control, it is important to include *pfhrp2-*deleted and *pfhrp3-*deleted strain controls such as the Dd2 and HB3 culture strains, respectively. This is important because several commonly used PCR assays have been shown to amplify the paralogous *pfhrp2* or *pfhrp3* genes under some conditions because of sequence homology in exon 1 of both genes [30].
4. Use care when conducting nested assays or evaluating low-parasite-density samples. Semi-nested and nested PCR can offer higher sensitivity for amplification of *pfhrp2* and *pfhrp3* from samples with low parasite densities. However, these approaches also increase the risk of cross-contamination and complicate laboratory workflows. Instead, many recent laboratory analyses have used a single round of PCR with 45 cycles instead of nested PCR [30, 32–35], and used a parasite density cutoff to select samples with sufficient target concentration to reduce the risk of false-negative results [36]. This approach works well in studies of symptomatic individuals, as parasite densities in symptomatic patients are generally high and give good PCR results with a single round PCR. This is also consistent with the WHO recommendations to prioritize gene deletion surveillance in symptomatic patients and enables assessment of false-negative RDT prevalence during case management.
5. Ensure adequate parasite DNA template is present before making deletion calls. Final *pfhrp2/3* deletion calls should only be made when PCR fails to amplify either exon of these genes but successfully amplifies at least two single-copy genes (using assays with the same number of amplification cycles). In addition, requiring a parasite density of ≥ 100 parasites/µL reduces the risk of unintentional misclassification of deletions due to PCR failure at low parasite DNA concentration. Including a positive control at low parasite density near the proposed threshold on each plate also provides a control for PCR sensitivity.
6. Verify assays before applying them to field samples. As with all molecular assays, it is essential to verify the performance of any *pfhrp2* or *pfhrp3* assay in each laboratory utilizing the assay. Differences in laboratory reagents, users, and equipment can affect PCR performance, particularly at low parasite density concentrations. At a minimum, each lab should first verify their *pfhrp2/3* PCR assays using replicates of serially diluted, well-characterized *P. falciparum* DNA. For example, an experiment including five replicates each of *pfhrp2-*deleted (ex: Dd2), *pfhrp3-*deleted (ex: HB3), and *pfhrp2/3-*intact (ex: 3D7) strain DNA at 100,000, 10,000, 1000, 100, 10, and 1 parasites/µL each will provide insights into the assays’ limits of detection and repeatability at different parasite densities. The results of assay verification could bolster confidence of results presented in publications, especially the estimated lower detection limit and rationale for selecting parasite density threshold for deletion calling.

### Multiplex real-time PCR

Recently developed multiplex real-time PCR assays for *pfhrp2/3* genes offer opportunities to improve throughput and reduced costs compared to conventional PCR and are outlined in Table 2. The primer/probe sites for the *pfhrp2/3* genes in conventional and qPCR assays are outlined in the Additional file 1. These probe-based assays can simultaneously amplify *pfhrp2*, *pfhrp3*, another *Plasmodium* gene, and an internal control gene (usually a human housekeeping gene). Existing multiplex *pfhrp2/3* real-time PCR assays target exon 1–2 and/or exon 2 using slightly different primer and probe targets, producing different amplicon sizes. In addition to *pfhrp2/3,* the three assays published to date target different parasite genes: *pfldh* [37], *pfrnr2e2* [38], and *cytb* [39]. While *pfldh* and *pfrnr2e2* are single-copy genes, *cytb*, (like the 18S rRNA gene which has 4–8 copies/genome)*,* is a multicopy gene (30–100 copies per genome) [40] and is expressed in the *P. falciparum* mitochondria. Due to differences in limits of detection, use of multicopy genes to verify enough parasite DNA template increases the risk of false deletion calls, particularly at low parasite density, and is discouraged.

**Table 2:** qPCR assays: primer positions, parasite reference target, human gene and amplicon size

qPCR assay initial report (reference)	*pfhrp2/3* target position	Parasite reference gene	Internal control	Amplicon size (base pairs)
Grignard et al. [37]	Exon 2	*pfldh*	Human *TUBB*	*pfhrp2*: 98 *pfhrp3*: 84
Kreidenweiss et al. [39]	Exon 2	*pfcytb*	Parasite *pfbtub*	*pfhrp2*: 78 *pfhrp3*: 79
Schindler et al. [38]	Exon 1–2	*pfrnr2e2*	None	*pfhrp2*: 286 *pfhrp3*: 289

Important considerations when conducting *pfhrp2/3* genotyping using multiplex real-time PCR:

1. Verify assays before applying them to field samples. The *pfhrp2/3* multiplex real-time PCR assays should first be verified in the performing laboratory, using well-characterized laboratory strain positive controls or samples with known *pfhrp2/3* status before applying them to unknown samples.
2. Always determine an assay’s reliable limit of detection in laboratory utilizing the assay. Through serial dilution series of purified DNA from known parasite densities, it is important to determine the limits of detection of the real-time PCR assays in the laboratory to ensure that they are not affected by operator or reagent differences.
3. Carefully determine the threshold line and the cycle threshold (Ct) cut-off value for negative results. This fluorescence threshold after a certain number of PCR cycles should be greater than the NTC on each assay plate and higher than the assay’s limit of detection. For example, analysis has often been restricted to 35 cycles to avoid the risk of spurious signal resulting from prolonged cycling time. Any PCR experiment with no florescence signal in the human gene target or a high florescence signal (low Ct) in the NTC (≤ 35) should be invalidated and repeated.
4. Evaluate sample integrity using human housekeeping genes. Use human housekeeping gene amplification as a guide to decide whether a sample is adequate for interpretation (positive human DNA signal) or invalid (no human DNA signal) and in need of repeating.
5. Include appropriate controls. Include lab culture controls with single gene deletions on every assay plate: *pfhrp2*-deleted (ex: Dd2 strain) and *pfhrp3*-deleted (ex: HB3 strain). Use of only one positive control with double deletion is not optimal as cross-binding of primers cannot be monitored. In addition, include an NTC and *pfhrp2/3-*intact positive controls such as the *P. falciparum* international DNA standard PfINT [41], or a well-characterized *P. falciparum* wild-type control with known parasitaemia.
6. Deletion calls in multiclonal infection: Ensure the PCR efficiency for all targets is similar and adjust thresholds to normalize cycle threshold values across targets. Confirm the Ct value for the standard positive control (preferably PfINT) is set in such a way that it is the same for the reference gene, *pfhrp2*, and *pfhrp3* before beginning to analyze the data for deletions in multiclonal infections. After optimization of PCR efficiency and careful adjustment of the Ct values of all targets, deletions in multiclonal infection where deleted parasites are the predominant strain can usually be called if the Ct difference between the reference (e.g., *pfldh*) and *pfhrp2* or *pfhrp3* is ≥ 3.

Real-time PCR assays that include a single-copy parasite reference gene target such as *pfldh* and *pfrnr2e2* are better suited for detecting *pfhrp2/3* deletions in multiclonal infection, particularly when the amplification efficiency of the reference gene is similar to that of *pfhrp2/3*. Current real-time PCR assays report detection of *pfhrp2/3-*deleted strains present at as low as 1% (minor clones) and as high as 80% (major clones) frequency in multiclonal infections [37]. However, the ability to detect deletions in multiclonal infections may vary between laboratories, and it is important to verify the performance of the real-time PCR assay using mixtures of laboratory strain DNA or well-characterized multiclonal infections in the performing laboratory before using for this purpose as a surveillance tool.

Multiplex *pfhrp2/3* PCR assays which include internal amplification controls do not have as great of a need for replicate wells or runs. To ensure confidence in deletion calls, samples with no human DNA signal or Ct value > 30.0 should be repeated. It would also be suggested to repeat the assay on all samples with initial PCR evidence of a deletion.

### Digital PCR

Digital PCR (dPCR) is another emerging technology that can be used to detect *pfhrp2/3* gene deletions. In this method, reactions are partitioned as chambers in microfluidics-based dPCR or as droplets in droplet digital PCR (ddPCR). Because reactions are partitioned to approximately 20,000 droplets and amplification occurs in a single partition, dPCR is suitable for absolute quantification of DNA targets, including detection of *pfhrp2/3* deletions in multiclonal infections [42]. Different studies have shown that quantification of target DNA using dPCR is affected by droplet volume and this in turn varies by manufacturer and laboratory [43]. Droplet volume has also been shown to be affected by the type of mastermix used [44]. Research on suitability of dPCR for detection of *pfhrp2/3* deletions should include minimum information on limit of detection, repeatability, reproducibility, droplet volume, mastermix (supermix), and analytical and diagnostic sensitivity. For groups looking to use these new assays within their laboratory, diagnostic sensitivity and specificity as well as quantification accuracy should be validated against conventional PCR and/or real-time qPCR to assure comparable performance.

### Next-generation sequencing

The *pfhrp2* and *pfhrp3* genes are located near the subtelomeric regions of chromosome 8 and 13, where genomic rearrangement including deletions commonly occur. These deletion events often involve large (~ 20 kb) chromosomal fragments that contain many genes, including *pfhrp2* and *pfhrp3* [45, 46]. Prior to whole-genome sequencing (WGS) being readily available, a strategy to examine the presence/absence of genes flanking *pfhrp2* and *pfhrp3* was used, providing supporting evidence for chromosomal deletions around the *pfhrp2* and *pfhrp3* location [14, 47]. Sanger sequencing of amplified *pfhrp2* and *pfhrp3* fragments has also been used to characterize partial deletions that occur within these genes [26], and to understand sequence structure and genetic diversity of these genes in gene-intact parasite population [29, 48].

In recent years, WGS has been used to confirm gene deletions on chromosomes and characterize deletion breakpoints on re-arranged chromosomes. *Plasmodium falciparum* WGS involves sequencing individual DNA molecules in a sample using one of several sequencing platforms, most commonly using short sequencing reads that are then mapped to a reference genome, or de novo assembled into contiguous sequences by chromosome. This approach provides opportunities to identify or confirm *pfhrp2/3* deletions, characterize *pfhrp2* and *pfhrp3* deletion patterns within genome sequence (ex: size, location of breakpoints), and study the genetic diversity of deleted strains [20, 46, 49–51]. However, the highly complex and repetitive nature of the *P. falciparum* subtelomere makes it challenging to assemble the regions where *pfhrp2* and *pfhrp3* reside on chromosomes 8 and 13, respectively. As a result, most *P. falciparum* genomic studies exclude these regions from analysis. Long-read sequencing approaches like those offered by PacBio or Oxford Nanopore Technologies have the potential to overcome these challenges, but their use for subtelomere and telomere assembly to date has been largely limited to culture-adapted parasites [11].

A recently developed amplicon-based deep sequencing approach uses molecular inversion probes (MIPs) to amplify hundreds of targets spanning *pfhrp2*, *pfhrp3*, and their flanking genes in a single reaction [36]. When applied to samples collected during a large *pfhrp2/3* deletion survey in Ethiopia, this approach enabled cost-effective, high-throughput mapping of deletion breakpoint regions and identifying genetic signatures of evolutionary selection favoring *pfhrp2/3-*deleted parasites. However, other methods such as conventional or real-time PCR are better suited for initial deletion screening to estimate prevalence, as MIP capture is inconsistent at lower parasite densities (< 1000 parasites/µL) but provide more detailed characterization of the deletions or parasite strains.

### Practical considerations for reference laboratories and testing in resource-limited settings

Capacity building and translation of these technologies to malaria endemic settings is critical for long-term sustainability of these surveillance efforts, timely data collection and reporting of results, and buy-in of national stakeholders. However, to the extent possible this capacity should be an add-on to existing non-malaria related efforts to use molecular based analysis for infectious diseases surveillance and/or case management purposes. The necessity for established lab capacity supporting cross-disease applications has become starkly apparent during the recent 2014–2016 west Africa Ebola epidemic [52], and the current SARS-CoV-2 pandemic [53, 54]. Similarly, high throughput laboratory based immunoassays that can screen for malaria antigens can also be utilized for detection of other proteins—antigens from other pathogens, human antibodies, or cytokines and immune factors [7]. In the same manner, technologies for nucleic acid amplification, detection, and sequencing are not specific for *pfhrp2/3* and can support other malaria and infectious disease surveillance activities.

While building integrated laboratory infrastructure and capacity should be a common goal, practical limitations may exist in different settings that prevent the use of advanced platforms. However, the most advanced technologies available are not necessarily required for *pfhrp2/3* deletion surveillance and confirmation of gene absence. Practically, the two-RDT testing algorithm comparing reactivity with HRP2 and Pf-pLDH test lines proposed in the WHO protocol could be instituted anywhere, regardless of laboratory capacity [17]. Additionally, testing of blood specimens by RDT can be done at point-of-care, or blood saved under cold storage for later assaying of HRP2 by RDT in another setting. This pragmatic and economical screening of symptomatic persons can provide strong phenotypic evidence for *P. falciparum* infections not producing HRP2 and HRP3 antigens and requiring molecular interrogation to elucidate *pfhrp2/3* genotype [18, 55, 56].

Amplification of the *pfhrp2/3* genes and assessment of results can be performed most simply using conventional or multiplex real-time PCR assays. Reagent and DNA storage for these assays only requires 4° and − 20° cold units, and thermocyclers or real-time PCR machines simply require a reliable source of electricity. Readouts and data interpretation of agarose gel and amplification curve results are not complex and produce a binary positive/negative result for amplification of a gene target. However, appropriate interpretation of results can only be accomplished with proper controls included with each assay plate or reaction and careful validation of assays prior to their use on clinical samples. Using proper laboratory workflows and controls as outlined above, a scientist can have high confidence in gene amplification results. In general, amplification of other single-copy *P. falciparum* genes and inclusion of assay wells containing an NTC and well-characterized *P. falciparum* strains with *pfhrp2/3* deletions are required [27].

Validation of new assays, or verification of performance of these assays in other laboratories, is critical before specimen screening of unknowns begins and mandatory before estimates regarding *pfhrp2/3* deletion can be made. Considerations should be made for assay repeatability and the estimated level of antigen or DNA the assay would be able to detect. Recombinant *Plasmodium* antigens are available for immunoassay validation, and true negative blood can be obtained from persons not residing in an endemic setting [21]. A defined panel consisting of known *P. falciparum* genotypes (culture or field isolates) can assist in comparison of DNA assay performance among laboratories and verifying the assay is fit-for-use in a setting. For molecular assays, the WHO offers a malaria nucleic acid amplification Malaria Molecular External Quality Assurance program (WHO Malaria NAAT EQA) that enables careful monitoring of PCR performance and access to well-characterized *Plasmodium* samples, including *pfhrp2/3* deleted parasites [57].

### Current gaps in *pfhrp2* deletion surveillance that laboratory data and deployment of new laboratory technologies need to overcome

Multiple factors that can lead to incorrect *pfhrp2/3* deletion genotyping results are outlined in Table 3. When more than one *P. falciparum* strain infects a single individual, the presence of parasites with *pfhrp2* (and/or *pfhrp3*) deletions can be masked. Antigen detection assays are indifferent to which *P. falciparum* genotype is producing the HRP2 (or other) antigens, thus if enough HRP2/HRP3 is in the blood, RDT or laboratory-based antigen tests will return a positive result regardless of whether one strain has a deletion of *pfhrp2* and/or *pfhrp3* [21, 58]. Similarly, conventional PCR assays will amplify a gene target if it is present, regardless of whether it is from the dominant *P. falciparum* strain or a minor strain. For this reason, if a HRP2-producing parasite exists within a multiclonal infection, it would potentially mask the presence of the *pfhrp2*-deleted strain using phenotypic or genotypic assays [24]. Therefore, in high-transmission areas where mixed-strain infections are common, the true prevalence of gene deletions in the *P. falciparum* population is likely to be underestimated. However, sufficient HRP2 in blood would still elicit a positive HRP2-RDT, thus providing appropriate case management regardless of how many (or which) genotypes exist within a patient. Additionally, WHO-recommended thresholds for switching to non-HRP2 diagnostics are based on a percentage of false-negative HRP2-RDTs caused by *pfhrp2/3* deletions, not a percentage of how many persons harbor an infection with a *pfhrp2-*deleted *P. falciparum* genotype [17].

**Table 3:** Different scenarios affecting the estimates for true *pfhrp2/3* deletion prevalence

Scenario	Implications for antigen detection	Implications for DNA detection	Implications for prevalence estimates and clinical impact	References
Multiclonal infection with wild-type and deleted *P. falciparum*	Deleted parasites not detected even though antigen is detected	Deleted parasites not detected by conventional PCR, though possible to detect using multiplex real-time or dPCR	Underestimate true gene deletion prevalence, though no clinical impact or effect on false-negative RDT prevalence	[24, 37, 38]
Residual HRP2 from previous *P. falciparum* infection, and current infection with deleted *P. falciparum*	Positive result regardless of current *P. falciparum* infection status	No effect	Underestimate true gene deletion prevalence due to phenotypic positivity. No clinical impact	[59, 60]
Low-level and spatial heterogeneity of deletions	Results not representative	Results not representative	Incomplete data available to guide decision making	[61]
Seasonal fluctuations in deletion prevalence	Results not representative	Results not representative	Incomplete data available to guide decision making	[25]

Since the development of lateral-flow RDTs, the duration of HRP2 antigenaemia has been shown to outlast *P. falciparum* parasitaemia for weeks to months [6, 59, 62, 63], though this is not observed for other *Plasmodium* antigens [63, 64]. From a clinical perspective, this means an individual may test HRP2 positive due to a recently successfully treated *P. falciparum* infection, though the etiological cause of their current ailment may be something else. For surveillance of *pfhrp2/3* deletions, this phenomenon would have the greatest effect on antigenaemia as a proxy for *Plasmodium* presence and could mask a parasite infection with a deleted genotype. If the patient is truly infected with *P. falciparum* that does not express HRP2, but the antigen remains in the blood from a previous infection, both HRP2 RDTs and laboratory immunoassays may misclassify this infection as wild-type without suspicion of *pfhrp2/3* deletion. This scenario is likely to occur more during high-transmission periods and/or in holoendemic settings where HRP2 antigenaemia may persist for many months—resulting from mixed-strain *P. falciparum* infections [25].

The majority of areas investigated for the existence of *pfhrp2/3* deletions in the resident *P. falciparum* population have found encouraging results of very few, or no, *P. falciparum* infections with *pfhrp2/3-*deleted parasites and when *P. falciparum* infection is identified, high HRP2/HRP3 antigen levels which will elicit positive HRP2-RDTs [16, 18–22, 24, 34, 56, 65]. However, true representative surveys (or surveillance systems) to drive policy on malaria diagnostics at a national level need to involve representative study sites from multiple localities, and transmission settings each screening enrolling hundreds of suspected malaria cases participants [17]. Additionally, clonal expansion of deleted genotypes could potentially be very focal in an area [36], and these genotypes could be missed if those affected sites are not sampled.

## Conclusions

Increasing reports describe *P. falciparum* with deletion (or other null mutation) of the *pfhrp2* and *pfhrp3* genes in malaria-endemic countries. As these deletions are known to arise de novo in *P. falciparum* populations and have been found across the globe, it is reasonable to assume that parasites with these gene deletions exist in most parasite populations. The most important questions are not whether these deletions exist in parasite populations, but whether their prevalence is high enough to affect the reliability of HRP2-RDTs for detection of *P. falciparum* malaria diagnostic strategies in a given country. Low-level and spatially-heterogeneous patterns of parasites with deletions will require larger sample sizes to obtain accurate and precise estimates. Multiple laboratory strategies to support these efforts now exist to determine HRP2 phenotype and deletion genotype. Low-cost screening tools will help make large-scale surveys and surveillance more practical, and establishment of lab capacity in endemic counties is an essential goal to attain. The choice of laboratory strategy should be informed by local technical and logistical considerations and must prioritize rigor and reproducibility.

## Supplementary Information

**Additional file 1: Figure S1.** Target sequences of *pfhrp2 and pfhrp3* for most primers in exon1 and exon2 locations of the genes. **A** The entire *pfhrp2* and *pfhrp3* genes showing location of the primers and probes. **B** Primer sequence locations for nested PCR amplification of *pfhrp2* and *pfhrp3* [14]. Locations of primers and probes for qPCR amplification of *pfhrp2* and *pfhrp3*: **C** Grignard et al. [37]; **D** Kreidenweiss et al. [39]; **E** Schindler et al. [38]. **F** Genome location of the amplicon and locations of primers for one-step PCR amplification of *pfhrp2* [31].

## Data Availability

Not applicable.

## Abbreviations

Ct: Cycle threshold
ELISA: Enzyme-linked immunosorbent assay
HRP2: Histidine-rich protein 2
HRP3: Histidine-rich protein 3
LDH: Lactate dehydrogenase
MIP: Molecular inversion probe
NTC: No-template control
PCR: Polymerase chain reaction
RDT: Rapid diagnostic test
WGS: Whole-genome sequencing
WHO: World Health Organization
