# Correction: Screening strategies and laboratory assays to support *Plasmodium falciparum* histidine-rich protein deletion surveillance: where we are and what is needed

**DOI:** 10.1186/s12936-022-04281-9

**Published:** 2022-09-14

**Authors:** Khalid B. Beshir, Jonathan B. Parr, Jane Cunningham, Qin Cheng, Eric Rogier

**Affiliations:** 1 Faculty of Infectious Diseases, London School of Hygiene and Tropical Diseases, Keppel Street, London, WC1E 7HT UK; 2 Division of Infectious Diseases and Institute for Global Health and Infectious Diseases, University of North Carolina at Chapel Hill, Chapel Hill, NC 27599 USA; 3 Global Malaria Programme, World Health Organization, Geneva, Switzerland; 4 Drug Resistance and Diagnostics, Australian Defence Force Malaria and Infectious Disease Institute, Brisbane, Australia; 5 QIMR Berghofer Medical Research Institute, Brisbane, Australia; 6 Division of Parasitic Diseases and Malaria, Center for Global Health, Centers for Disease Control and Prevention, Atlanta, GA 30029 USA

## Correction: Malaria Journal (2022) 21:201 10.1186/s12936-022-04226-2

Following publication of the original article [1], the authors requested the following corrections:

- Page 6 (of the PDF)—This sentence needs revision: “In addition to pfhrp2/3, the three assays published to date target different parasite genes: pfldh [37], pfrnr2e2 [38], and cytb [39]”. In this sentence, change “cytb” to “pfbtub”.
- Page 6 (of the PDF)—This sentence needs revision: “While pfldh and pfrnr2e2 are single-copy genes, cytb, (like the 18S rRNA gene which has 4–8 copies/genome), is a multicopy gene (30–100 copies per genome) [40] and is expressed in the *P. falciparum* mitochondria.”. This should be split into two sentences, and should read: “The pfldh, pfrnr2e2, and pfbtub genes are all single-copy which make them the most appropriate as references for reporting pfhrp2/3 genotype. Genes like cytb (similar to the 18S rRNA gene, which has 4–8 copies/genome), is a multicopy gene (30–100 copies per genome) [40] and is expressed in the *P. falciparum* mitochondria.”
- Page 6 (of the PDF)—Table 2. Within the table in the second row, the parasite reference gene is listed as ‘pfcytb’ and internal control is ‘Parasite pfbtub’. The reference gene should be listed as ‘Parasite pfbtub’ and internal control as ‘None’.
