# Supplementary material for: Screening strategies and laboratory assays to support Plasmodium falciparum histidine-rich protein deletion surveillance: where we are and what is needed
Source: Malar J. 2022 Jun 24;21:201. doi: 10.1186/s12936-022-04226-2 (PMC9233320; doi:10.1186/s12936-022-04226-2)
Supplement: Supplementary file 1 — Additional file 1: Figure S1. Target sequences of pfhrp2 and pfhrp3 for most primers in exon1 and exon2 locations of the genes. A The entire pfhrp2 and pfhrp3 genes showing location of the primers and probes. B Primer sequence locations for nested PCR amplification of pfhrp2 and pfhrp3 [14]. Locations of primers and probes for qPCR amplification of pfhrp2 and pfhrp3: C Grignard et al. [37]; D Kreidenweiss et al. [39]; E Schindler et al. [38]. F Genome location of the amplicon and locations of primers for one-step PCR amplification of pfhrp2 [31]. [file 12936_2022_4226_MOESM1_ESM.docx]

**Additional File**


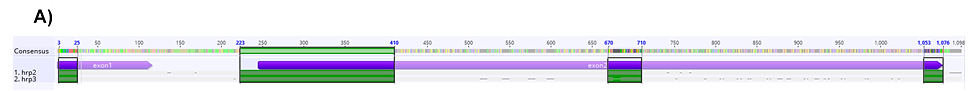


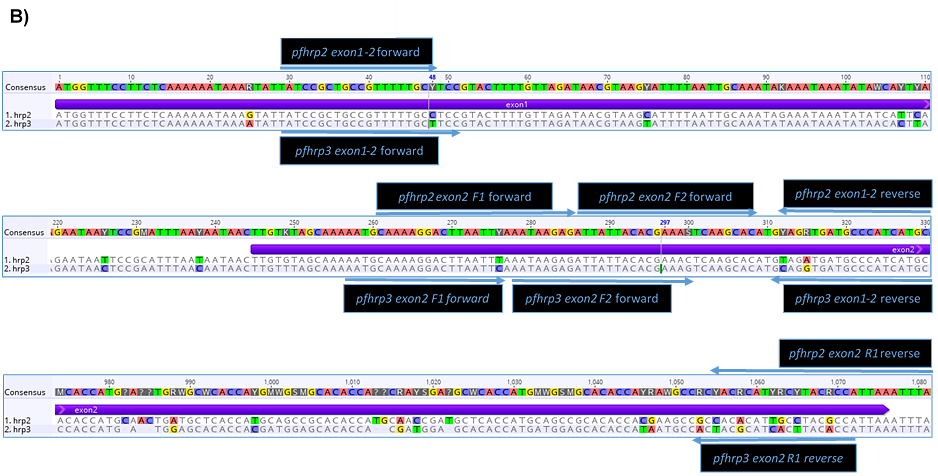


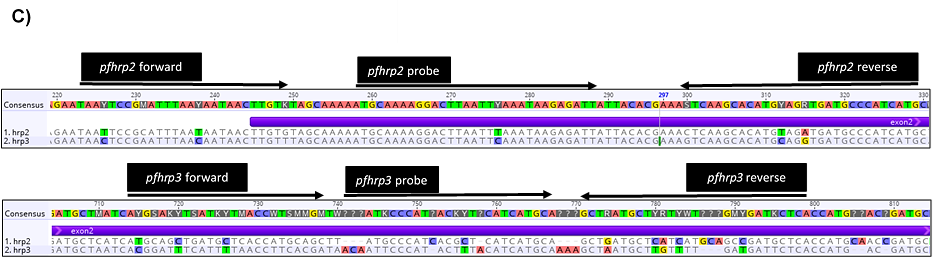


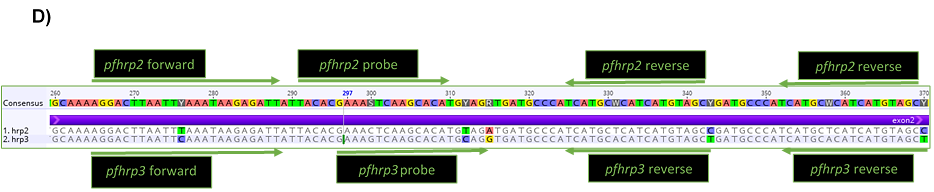


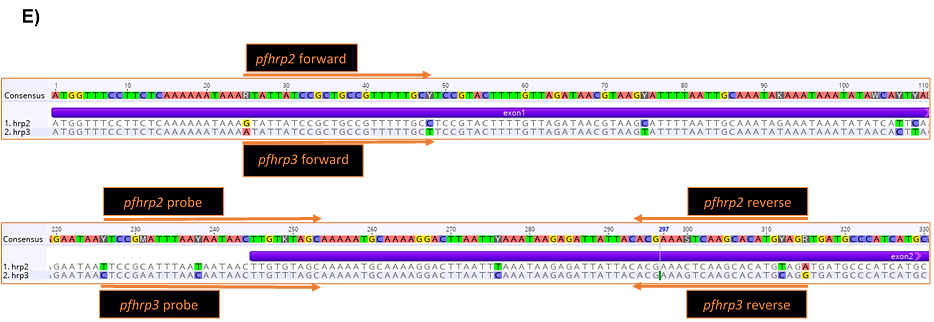


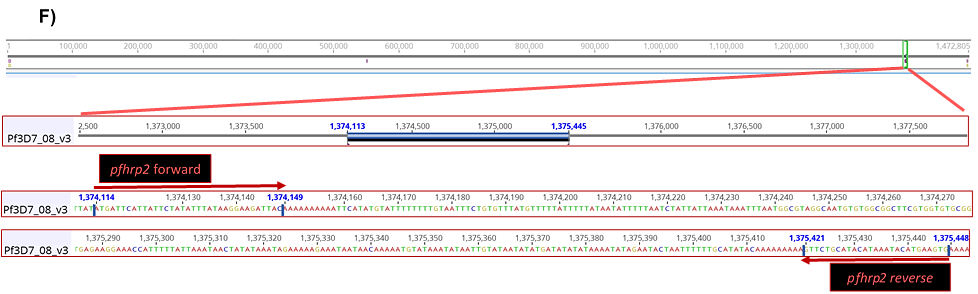


**Additional File.** Target sequences of *pfhrp2 and pfhrp3* for most primers in exon1 and exon2 locations of the genes. (**A**) The entire *pfhrp2* and *pfhrp3* genes showing location of the primers and probes. (**B**) Primer sequence locations for nested PCR amplification of *pfhrp2* and *pfhrp3* (Gamboa, *et al*, 14). Locations of primers and probes for qPCR amplification of *pfhrp2* and *pfhrp3*: (**C**)(Grignard, *et al*, 37); (**D**)(Kreidenweiss, *et al*, 39); (**E**)(Schindler, *et al*, 38). (**F**) Genome location of the amplicon and locations of primers for one-step PCR amplification of *pfhrp2* (Jones, *et al*, 31).
